# JASPAR 2024: 20th anniversary of the open-access database of transcription factor binding profiles

**DOI:** 10.1093/nar/gkad1059

**Published:** 2023-11-14

**Authors:** Ieva Rauluseviciute, Rafael Riudavets-Puig, Romain Blanc-Mathieu, Jaime A Castro-Mondragon, Katalin Ferenc, Vipin Kumar, Roza Berhanu Lemma, Jérémy Lucas, Jeanne Chèneby, Damir Baranasic, Aziz Khan, Oriol Fornes, Sveinung Gundersen, Morten Johansen, Eivind Hovig, Boris Lenhard, Albin Sandelin, Wyeth W Wasserman, François Parcy, Anthony Mathelier

**Affiliations:** Centre for Molecular Medicine Norway (NCMM), Nordic EMBL Partnership, University of Oslo, 0318 Oslo, Norway; Centre for Molecular Medicine Norway (NCMM), Nordic EMBL Partnership, University of Oslo, 0318 Oslo, Norway; Laboratoire Physiologie Cellulaire et Végétale, Univ. Grenoble Alpes, CNRS, CEA, INRAE, IRIG-DBSCI-LPCV, 17 avenue des martyrs, F-38054, Grenoble, France; Centre for Molecular Medicine Norway (NCMM), Nordic EMBL Partnership, University of Oslo, 0318 Oslo, Norway; Centre for Molecular Medicine Norway (NCMM), Nordic EMBL Partnership, University of Oslo, 0318 Oslo, Norway; Centre for Molecular Medicine Norway (NCMM), Nordic EMBL Partnership, University of Oslo, 0318 Oslo, Norway; Centre for Molecular Medicine Norway (NCMM), Nordic EMBL Partnership, University of Oslo, 0318 Oslo, Norway; Laboratoire Physiologie Cellulaire et Végétale, Univ. Grenoble Alpes, CNRS, CEA, INRAE, IRIG-DBSCI-LPCV, 17 avenue des martyrs, F-38054, Grenoble, France; Center for Bioinformatics, Department of Informatics, University of Oslo, Oslo, Norway; MRC London Institute of Medical Sciences, Du Cane Road, London W12 0NN, UK; Institute of Clinical Sciences, Faculty of Medicine, Imperial College London, Hammersmith Hospital Campus, Du Cane Road, London W12 0NN, UK; Division of Electronics, Ruđer Bošković Institute, Bijenička cesta, 10000 Zagreb, Croatia; Centre for Molecular Medicine Norway (NCMM), Nordic EMBL Partnership, University of Oslo, 0318 Oslo, Norway; Stanford Cancer Institute, Stanford University School of Medicine, Stanford, CA 94305, USA; Centre for Molecular Medicine and Therapeutics, Department of Medical Genetics, BC Children's Hospital Research Institute, University of British Columbia, 950 W 28th Ave, Vancouver, BC V5Z 4H4, Canada; Center for Bioinformatics, Department of Informatics, University of Oslo, Oslo, Norway; Center for Bioinformatics, Department of Informatics, University of Oslo, Oslo, Norway; Center for Bioinformatics, Department of Informatics, University of Oslo, Oslo, Norway; Department of Tumor Biology, Institute for Cancer Research, Oslo University Hospital, 0424 Oslo, Norway; MRC London Institute of Medical Sciences, Du Cane Road, London W12 0NN, UK; Institute of Clinical Sciences, Faculty of Medicine, Imperial College London, Hammersmith Hospital Campus, Du Cane Road, London W12 0NN, UK; Department of Biology and Biotech Research and Innovation Centre, University of Copenhagen, Ole Maaløes Vej 5, DK2200 Copenhagen N, Denmark; Centre for Molecular Medicine and Therapeutics, Department of Medical Genetics, BC Children's Hospital Research Institute, University of British Columbia, 950 W 28th Ave, Vancouver, BC V5Z 4H4, Canada; Laboratoire Physiologie Cellulaire et Végétale, Univ. Grenoble Alpes, CNRS, CEA, INRAE, IRIG-DBSCI-LPCV, 17 avenue des martyrs, F-38054, Grenoble, France; Centre for Molecular Medicine Norway (NCMM), Nordic EMBL Partnership, University of Oslo, 0318 Oslo, Norway; Center for Bioinformatics, Department of Informatics, University of Oslo, Oslo, Norway; Department of Medical Genetics, Institute of Clinical Medicine, University of Oslo and Oslo University Hospital, Oslo, Norway

## Abstract

JASPAR (https://jaspar.elixir.no/) is a widely-used open-access database presenting manually curated high-quality and non-redundant DNA-binding profiles for transcription factors (TFs) across taxa. In this 10th release and 20th-anniversary update, the CORE collection has expanded with 329 new profiles. We updated three existing profiles and provided orthogonal support for 72 profiles from the previous release's UNVALIDATED collection. Altogether, the JASPAR 2024 update provides a 20% increase in CORE profiles from the previous release. A trimming algorithm enhanced profiles by removing low information content flanking base pairs, which were likely uninformative (within the capacity of the PFM models) for TFBS predictions and modelling TF-DNA interactions. This release includes enhanced metadata, featuring a refined classification for plant TFs’ structural DNA-binding domains. The new JASPAR collections prompt updates to the genomic tracks of predicted TF binding sites (TFBSs) in 8 organisms, with human and mouse tracks available as native tracks in the UCSC Genome browser. All data are available through the JASPAR web interface and programmatically through its API and the updated Bioconductor and pyJASPAR packages. Finally, a new TFBS extraction tool enables users to retrieve predicted JASPAR TFBSs intersecting their genomic regions of interest.

## Graphical Abstract

**Graphical Abstract:**
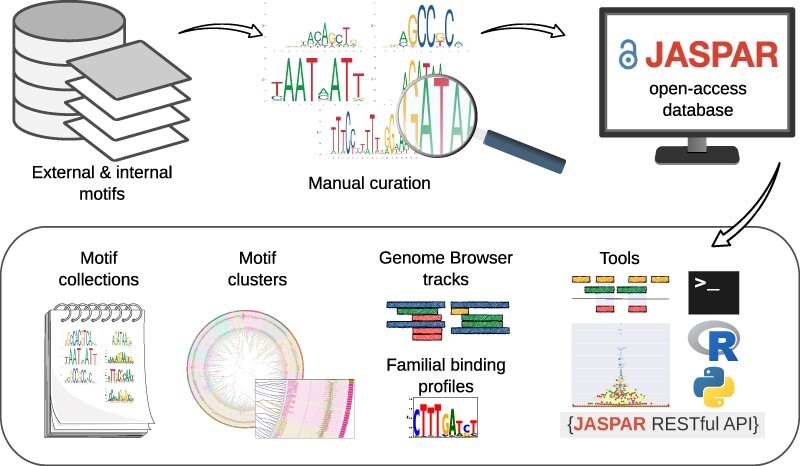

## Introduction

Transcriptional gene regulation is mediated through the interactions of specific regulatory proteins, notably transcription factors (TFs), with *cis-*regulatory genomic elements, including promoters and enhancers (1). TFs are a broad class of proteins that regulate and mediate transcription; they can be classified as general TFs, sequence-specific DNA binding TFs, or transcriptional co-regulators (we refer the readers to (2) for more details). Within this report, we limit the application of the TF term to the subset that engages with DNA in a sequence-specific manner via DNA binding domains (DBDs) (1,2). The sequence-specific binding of TFs at *cis*-regulatory elements occurs at TF binding sites (TFBSs), delineated genomic regions that are typically 6–20 bp in length (3). TFs can be classified into structurally related families based on their DBDs. TFs with DBDs from the same structural family tend to recognise similar DNA sequence motifs, except for zinc finger proteins (4,5). Although several biochemical and genome-wide assays exist to assess TF-DNA affinities and detect TFBSs, these assays cannot be performed for all TFs in all cell types and biological conditions. Thus, computational approaches and models for TF binding remain critical. Aside from prediction of TFBS, such models can also be used as part of other analyses, such as enrichment of TFBSs in sets of promoters or enhancers, prediction of impacts of mutations in non-coding regions and guided *in vitro* mutagenesis (6,7).

Position frequency matrices (PFMs) remain the most common computational representation of TF-DNA interactions. PFMs are quantitative summaries of the DNA-binding preferences of a given TF, tallying the frequency of each nucleotide at each position of an aligned set of TFBSs and storing this information in a matrix form. These matrices can be converted into probabilistic matrices termed position weight matrices (PWMs) (8). The primary function of PWMs is to model the binding affinity or probability of interaction between a TF and a DNA sequence (8). As such, PWMs are used to predict TFBSs within any DNA sequence. To systematically explore the functional effects stemming from the binding process these models describe, it is necessary to have an extensive compilation that mirrors their diversity. To address this issue, multiple database solutions have been developed to collect and store PFMs, such as JASPAR (9), CIS-BP (10), and HOCOMOCO (11).

JASPAR is a regularly maintained, open-access database that stores manually curated high-quality DNA binding profiles of TFs as PFMs. For the past two decades, JASPAR has consistently upheld its core principles of (i) providing high-quality TF binding profiles, (ii) fostering open access, and (iii) ensuring ease of use. These core principles drove its evolution and growth and contributed to JASPAR’s usefulness in the scientific community studying gene transcription regulation. JASPAR is now a standard resource in computational regulatory genomics (Supplementary Figure S1).

Central to JASPAR’s mission is to provide the community with an extensively curated, non-redundant collection of profiles from published resources and literature. This effort produces a CORE collection of profiles where at least two orthogonal experimental supports validate each entry. The quality-control process grew along with JASPAR’s expansion, introducing tools to aid curation. For example, we rely on the inference tool introduced in 2016 to support TF binding based on the similarity of DBDs between TFs (12). In 2020, we complemented the JASPAR CORE collection with the UNVALIDATED collection to reflect the increase in profiles derived from the broader use of high-throughput sequencing methods for which independent validation is yet to be produced (13). In order to have credible profiles within the UNVALIDATED collection, we kept the notion of high quality by putting the profiles under rigorous curation, where we look at the enrichment for TFBSs close to ChIP-seq peak summits (14), among other criteria. Although the profiles in the UNVALIDATED collection are computationally sound, we explicitly inform the users that these profiles should be used cautiously due to a lack of orthogonal support.

JASPAR offered the first open-access TF-binding profiles database with a web interface enabling direct download of the PFMs collected. The ease of data access and download manifests another core principle of JASPAR: the active effort to promote open science. As open science initiatives emerged in this field over the years, we witnessed a growing tendency to integrate those various resources into an ecosystem where tools and repositories build upon one another. These efforts eventually produced a synergistic ecosystem aligned with FAIR principles (15). The mutual integration of these various resources benefited from their early interoperability with an active effort to share standards for data formats and the possibility to leverage their respective source data. This latter point relies on the open accessibility of the data, which JASPAR adopted as a design choice from its beginning, with all profiles made available as simple flat text files. An additional dimension to this ecosystem is the engagement with our user community in our effort to strengthen the growth and quality of JASPAR’s content. For instance, we manifested our engagement using Google Groups for Q&As and by introducing an online form enabling users to notify the JASPAR team directly about profiles to add, validate, or update (13).

From its inception, JASPAR provided a web interface catering to the needs of both wet and dry researchers, illustrating JASPAR’s emphasis on ‘ease of use.’ This principle translates into design choices for the JASPAR database, starting with the data organised in a simple schema trying to make one profile correspond to one TF or one dimeric complex (e.g. MYC::MAX) in one taxon, corresponding to the non-redundant aspect of JASPAR. However, this condition was later relaxed in 2020 to introduce binding variants (13), reflecting the possibility for some TFs to bind two or more distinct DNA motifs. This simple architecture makes JASPAR easy to engage for any user. We further provide different interfaces ranging from straightforward web-interface to programmatic access through various packages in Perl (16), Python (17), R/Bioconductor (18), and Ruby (12). Further catering to the community's needs, access to JASPAR data has been expanded to incorporate a platform- and language-independent interface through the recent introduction of the JASPAR RESTful API (19). JASPAR’s ease of use was further facilitated by introducing the new web-interface and including the ‘JASPAR dynamic tour’ in 2018, which guides users through the typical tasks and novel features of the JASPAR website (20).

The team behind JASPAR has continuously tried to encompass the current scope of the data produced in the field. Of note in this effort is the rapid expansion witnessed following the introduction of high-throughput sequencing assays such as ChIP-seq (21), DAP-seq (22), PBM (23), SMiLE-seq (24), and HT-SELEX (25), which accelerated the generation of datasets suitable for modelling TF-DNA interactions (26,27). This process, which started with vertebrates, eventually reached all taxa present in JASPAR. Faced with this expansion, we adapted the procedures and pipelines at the source of JASPAR, moving from the original manual survey of journals and subsequent construction of profiles directly from article tables or images to a systematic motif processing pipeline from online resources. This process was also fueled by the integration of data from ReMap (28), GTRD (29), and CIS-BP (4) directly into our pipelines, illustrating the merit of such open science efforts in consolidating the field as a whole again. Furthermore, the increasing number of profiles inferred across numerous taxa allowed new functionalities, such as interactive profile clustering trees (20) or archetypes (9), to assist users in interpreting individual profiles within a broader context.

Here, we present the 10th release of the JASPAR database, providing a substantial update and expansion of TF binding profiles in seven taxonomic groups. This update includes the addition of 329 profiles as PFMs, orthogonal support for 72 profiles stored in the previous release's UNVALIDATED collection (i.e. they are now part of the CORE collection), an update of three profiles and an update of the metadata for 241 profiles. Moreover, 182 new PFMs were added to the UNVALIDATED collection. This release further includes updates to the word clouds displaying enriched terms associated with TFs in the literature, further improvement of the structural classification of plant TF DBDs, updated native UCSC human and mouse genome tracks with TFBSs predicted from JASPAR TF binding profiles, and updates on the various JASPAR tools such as the TFBS enrichment tool, pyJASPAR and R/Bioconductor packages. We introduce a new motif trimming algorithm to remove flanking positions from PFMs with low information content. Finally, we provide a new TFBS extraction tool to perform extraction of predicted JASPAR TFBSs intersecting with an input set of genomic regions provided by users.

## Results

### Expansion and update of the TF binding profiles

**Figure 1.**
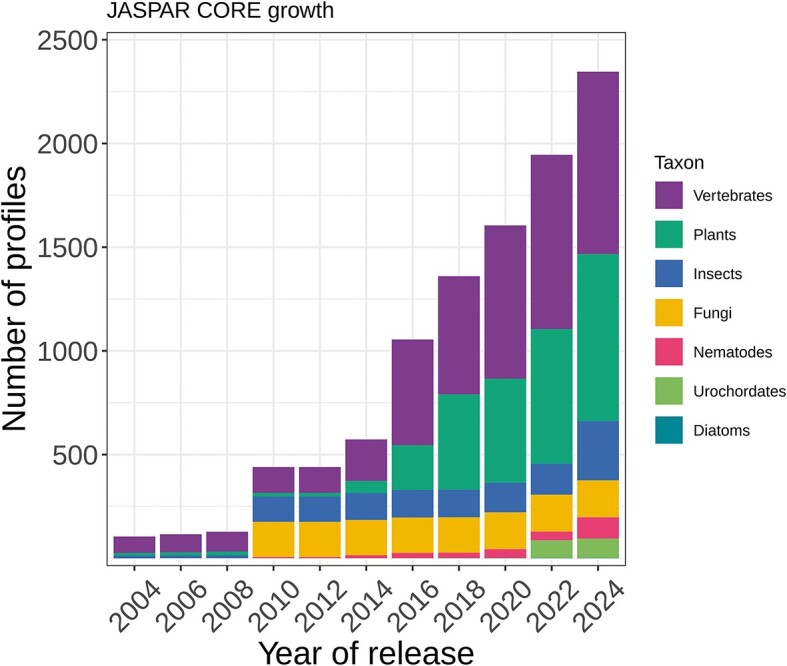
Overview of the growth of the number of profiles in JASPAR CORE collection across releases and taxons.

We retrieved TF binding profiles as PFMs from Lai *et al.* (30) for the PBM experiments, from CIS-BP (4) for TFs in insects, nematodes, and plants, from Bass *et al.* for worms (31), and from the UNVALIDATED collection of the JASPAR 2022 release (9) (Supplementary Table S1). We processed ChIP-seq, ChIP-exo, and DAP-seq datasets from GTRD (29) and ChIP-exo data from Lai *et al.* (30) using the RSAT *peak-motifs* tool (32) to identify enriched motifs (as PFMs) in the corresponding peak sets (Supplementary Table S1 and Supplementary Text for dataset and method details). Our expert curators manually selected the PFMs supported by orthogonal evidence from the literature to either add them to or update former TF binding profiles in the JASPAR CORE collection. The PFMs deemed high quality, but for which our curators did not find any orthogonal support in the literature were added to the JASPAR UNVALIDATED collection. We complemented the JASPAR CORE collection with 329 TF binding profiles and updated three existing profiles with new PFMs (Table 1 and Figure 1). We identified orthogonal support in the literature for 72 profiles previously stored in the JASPAR UNVALIDATED collection, promoting them to the CORE collection. Overall, the new JASPAR 2024 CORE collection represents a 20% increase in the number of profiles compared to the previous release (Supplementary Table S2). We augmented the JASPAR UNVALIDATED collection with 182 profiles (Supplementary Table S3). Finally, we updated the metadata associated with the profiles wherever possible (for 241 and 55 CORE and UNVALIDATED profiles, respectively) and removed 28 profiles (11 from the CORE collection and 17 from the UNVALIDATED collection) as these profiles were either redundant with other profiles, incorrectly supported by the literature, or associated with a protein not considered as a DNA-binding specific TF.

**Table 1.** Summary of the JASPAR 2024 update compared to the previous release

Taxonomic group in CORE collection	Non-redundant PFMs in JASPAR 2022	New non-redundant PFMs	Removed PFMs	Promoted PFMs (from UNVALIDATED to CORE)	Updated PFMs	Total non-redundant PFMs in JASPAR 2024
***Plants***	656	114	7	42	2	805
***Vertebrates***	841	19	1	20	1	879
***Urochordata***	86	-	-	8	-	94
***Insects***	150	135	1	2	-	286
***Nematodes***	43	61	1	-	-	103
***Fungi***	179	-	1	-	-	178
***Diatoms***	1	-	-	-	-	1
**CORE total**	**1956**	**329**	**11**	**72**	**3**	**2346**

The JASPAR 2024 release culminates with 2346 TF binding profiles in the CORE collection and 643 in the UNVALIDATED collection (Figure 1 and Supplementary Figure S2). In addition, we generated transcription factor flexible models (TFFMs; hidden Markov-based models capturing dinucleotide dependencies in TF–DNA interactions (33)) using all new CORE PFMs for which ChIP-seq data was available, resulting in 75 new TFFMs (45 for plants, 12 for vertebrates, 11 for insects, and 7 for nematodes). The JASPAR 2024 release compiles 1135 TFFMs (Supplementary Table S4). The web interface to access and visualise all profiles and metadata is accessible at https://jaspar.elixir.no, now hosted by ELIXIR Norway and recognised as a Norwegian bioinformatics service.

### Trimming of TF binding profiles

**Figure 2.**
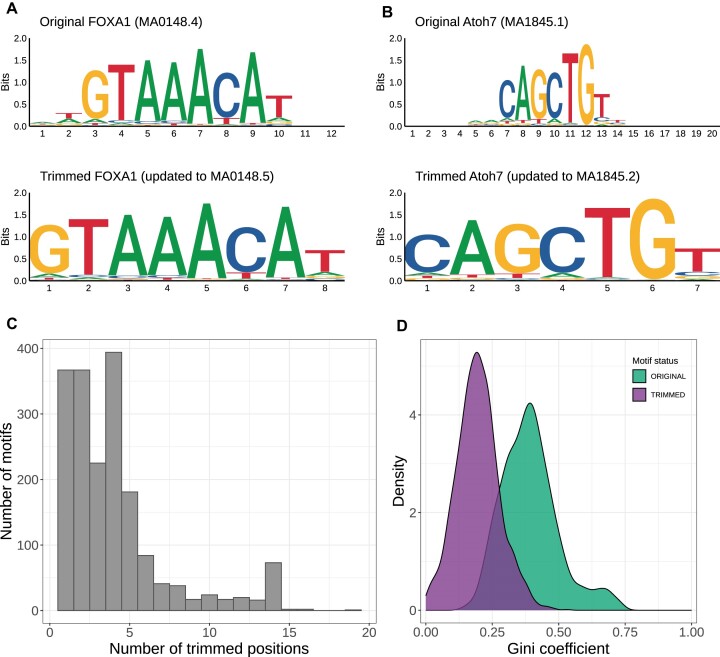
TF binding profile trimming. (A, B) Examples of trimmed PFMs for FOXA1 (**A**) and Atoh7 (**B**) TFs with the logos of the original PFMs at the top and the logos of the trimmed PFMs at the bottom. (**C**) The number of trimmed positions varied from 1 to 19 for PFMs originating from JASPAR 2022. (**D**) The distribution of Gini coefficients computed on the IC of each position in the original (green) and trimmed (purple) PFMs from JASPAR 2022 exhibits a concentration of informative positions in the updated PFMs.

Most TF binding profiles stored in JASPAR derive from computational *de novo* motif discovery tools applied to *in vitro* and *in vivo* data. The underlying algorithms sometimes report PFMs with low information content (IC) at the flanks (Figure 2A-B, top logos). The corresponding positions with low information content are likely uninformative (within the capacity of the PFM models) for predicting TFBSs and modelling TF-DNA interactions. We designed an algorithm to remove these uninformative flanking positions in the latest version of all the TF binding profiles available in the JASPAR CORE and UNVALIDATED collections (Supplementary Text for the detailed method). The bottom logos in Figures 2A-B illustrate case examples of the TF binding profile trimming algorithm results. The algorithm trimmed up to 19 positions (Figure 2C) in 1869 (1457 from the JASPAR 2022 CORE collection and 412 from the JASPAR 2022 UNVALIDATED collection) out of 2506 profiles. All newly curated profiles were trimmed by default. After trimming, the PFMs stored in JASPAR 2024 are 4–33 bp long (Supplementary Figure S3). As expected, the trimmed profiles concentrate on informative positions, as determined by Gini coefficients, which measure the inequality of values in a distribution (Figure 2D).

### Improved structural classification of plant TFs

Previous JASPAR versions have used TFClass as the reference structural classification for TF DBDs (34,35). However, this classification was built for mammal TFs and lacks many DBD types in plant genomes. This new JASPAR release benefited from the recent creation of a plant classification (Plant-TFClass) that includes 8 TF classes and 37 families absent from TFClass (36). We curated all entries in the JASPAR plant collection with this new classification.

### TF binding profile clusters, familial binding profiles, word clouds and genomic tracks

Beyond TF binding profiles, JASPAR provides several complementary features to the community to compare, analyse, and interpret genomic data in the context of transcriptional regulation of gene expression. Users can visualise the TF binding profiles' similarity in the CORE and UNVALIDATED collections through a radial tree. We updated the RSAT *matrix-clustering* tool (37) into a faster and expanded stand-alone version (https://github.com/jaimicore/matrix-clustering_stand-alone). We applied the tool to the PFMs stored in JASPAR to provide hierarchical clustering of the profiles in every taxon. To remove redundancy due to similar profiles, we computed familial binding profiles, which summarise similar profiles with a single PFM, by relying on hierarchical clustering (38). We followed the same methodology as introduced in the previous JASPAR release (9) to construct 408 familial binding profiles (155 for vertebrates, 52 for plants, 62 for fungi, 44 for nematodes, 76 for insects and 19 for urochordates). We provide all hierarchical clusters and familial binding profile summaries at https://jaspar.elixir.no/matrix-clusters.

In the JASPAR 2022 release, we introduced word clouds to summarise biological information associated with each TF. Specifically, the word clouds illustrate the significance of each word found in the abstracts associated with each TF by comparing their occurrences to those found in the abstracts of other TFs within the same taxon. For JASPAR 2024, we have created word clouds for newly added profiles and updated existing ones with up-to-date literature queries from PubMed.

We scanned the latest genome assemblies of eight species (*Arabidopsis thaliana, Caenorhabditis elegans, Ciona intestinalis, Danio rerio, Drosophila melanogaster, Homo sapiens, Mus musculus, and Saccharomyces cerevisiae*) with the latest version of all TF binding profiles from the corresponding taxon in the JASPAR CORE collection. This release includes both TF binding profiles for new TFs and trimmed profiles from the previous release to focus on informative positions. Upon comparing genome-wide TFBS predictions using JASPAR 2022 profiles with the corresponding trimmed profiles in JASPAR 2024, we found that most profiles yielded a comparable number of TFBS predictions. However, trimmed profiles predicted more TFBSs overall, with a few exceptions leading to a substantially increased number of predictions (Supplementary Figure S4). Additionally, we relied on the familial binding profiles to merge overlapping TFBSs predicted from similar PFMs. We provide users with the pre-computed TFBS prediction tracks for all TF binding profiles and familial binding profiles for genome visualisation and interpretation; the human and mouse TFBS tracks derived from TF binding profiles in the CORE collection are available as native tracks in the UCSC Genome Browser with prediction scores, logos, and TF names (39).

### JASPAR-associated tools

#### pyJASPAR and R/Bioconductor data package

Beyond the web interface and RESTful API, we provide programmatic access to the data stored in the JASPAR database. Specifically, the users can utilise the pyJASPAR Python package (https://github.com/asntech/pyjaspar) (40) and the JASPAR2024 R/Bioconductor data package (https://bioconductor.org/packages/JASPAR2024) to retrieve the data serverless. These packages allow for seamless integration of JASPAR data into Python and R workflows, providing the community with flexible and efficient means of programmatically retrieving and utilising JASPAR data for their research needs.

#### JASPAR TFBS enrichment tool

We previously introduced a command-line interface to perform TFBS enrichment analyses with JASPAR TFBS predictions in user-provided genomic regions (9). An update of the JASPAR TFBS predictions stored in the underlying LOLA databases for the enrichment tool accompanies the JASPAR 2024 release (41); users can find the LOLA databases on Zenodo at https://doi.org/10.5281/zenodo.8341374. Moreover, we now provide a Docker container for the JASPAR TFBS enrichment tool at https://hub.docker.com/r/cbgr/jaspar_tfbs_enrichment.

#### JASPAR TFBS extraction tool

This new JASPAR release comes with a new computational tool to extract predicted JASPAR TFBSs intersecting with a user-provided input set of genomic regions. TFBSs can be further filtered by providing TF names, JASPAR matrix IDs and TFBS score thresholds. The software is available as a command-line tool at https://bitbucket.org/CBGR/jaspar_tfbs_extraction and in a Docker container at https://hub.docker.com/r/cbgr/jaspar_tfbs_extraction.

## Conclusions and perspectives

For the 10th update of the JASPAR database, we expanded the JASPAR CORE collection by 20% (329 added and 72 upgraded profiles). The new profiles were introduced after manual curation, during which we curated 26 629 TF binding motifs obtained as PFMs or discovered from ChIP-seq/-exo or DAP-seq data. We also revised 2500 profiles from JASPAR 2022 to either promote them to the CORE collection, update the associated metadata, or remove them because of validation inconsistencies or poor quality. The insects and nematodes taxonomic groups received significant additions in the CORE collection (90% and 140% increase, respectively). Preparing this anniversary update, we focussed not only on expanding our profile collections but also on revisiting the quality of current motifs, especially renewing annotations for plant TF families and classes according to the Plant-TFClass (36) and searching for validation for profiles in UNVALIDATED collection.

The continuous expansion of the JASPAR database provides TF binding profiles for an increasing number of TFs from different organisms. With this release, the JASPAR CORE vertebrates collection presents a motif for 53% of the 1435 curated human TFs (58% of the 1118 orthologous mouse TFs) (2), 67% (64% of mouse orthologs) when adding profiles from the UNVALIDATED collection. The JASPAR CORE plants collection presents profiles for 32% of the 1717 reported *A. thaliana* TFs (42), 35% when including profiles from the UNVALIDATED collection. Another example is the JASPAR CORE insects collection, which provides a motif for 43% of the 628 TFs reported for *D. melanogaster* (43) and 50% when including UNVALIDATED profiles. A steady effort from the community to cover all TFs will be necessary to fill the remaining gap.

So far, the JASPAR database has stored and focused mostly on PFMs as the model of choice for TF-DNA interactions. We recognise that the PFMs stored in JASPAR assume nucleotide independence and do not consider the methylation status of nucleotides, which would require DNA methylation data and an expanded alphabet or specific representation (44–46). To account for successive nucleotide dependencies, we introduced transcription factor flexible models (TFFMs) into JASPAR for a set of profiles when data was available to compute them (12,33). Mostly based on convolutional neural networks, deep learning models are now considered state-of-the-art to accurately model data generated from genomic assays such as ChIP-seq, ChIP-nexus, or ATAC-seq (47–49). Some deep learning models have improved performance when initialising their convolutional filters with PWMs derived from JASPAR profiles (50,51), while many models assess derived patterns by comparison with JASPAR profiles (47,48,52). The high quality of the modelling and improved methods to interpret the deep learning models make them attractive to decipher the *cis-*regulatory code (53). With deep learning approaches becoming critical to studying TF-DNA interactions and discovering the regulatory grammar controlling gene transcription, models based on neural networks will potentially replace PFMs. Similarly to PFMs, deep learning models could be curated and stored in JASPAR. Work remains to incorporate such models in a manner that holds true to the JASPAR ease-of-use principle, consistent with observations from other bioinformatics applications (54,55). Software tools for scanning DNA sequences with diverse deep learning-based motif models are maturing (56), as are methods for understanding motif enrichment and/or combinatorics (51,57–60). In addition to refining efficient motif scanning tools, one important remaining step is to determine how to effectively handle context-specific models (e.g. specific cell types or tissues), as such models can capture motifs for cooperative TFs unavailable in other cell types (61). These next steps demand continued innovation for JASPAR in the years ahead.

While we are pleased with the first 20 years of impact by JASPAR, we recognise that genomics and bioinformatics demand constant observation of the road ahead. We expect that the growing use of intelligent systems to derive inference from large data will continue to accelerate in use. As represented by large language models and deep learning-based image generation technologies, we can expect that bioinformatics methods will increasingly seek to bridge molecular data with structured knowledge. Including word clouds in JASPAR represents an initial movement in this direction. However, ultimately we expect that the binding models will need to be complemented with advanced knowledge representation about TFs, either in the form of knowledge graphs (such as in (62,63)) and/or vectors describing contextual embeddings.

Since the beginning, JASPAR has grown to provide the community with a high-quality, easy-to-use resource that promotes open science. Over the years, JASPAR’s scale and scope faithfully accompanied the technological and scientific developments in the field. Striving to ensure the high quality of the database content throughout its continuous expansion meant regularly re-visiting the sourcing, processing, and presentation of JASPAR. This effort was further oriented towards maintaining JASPAR’s ease of use, incorporating functionalities and content deliberately to address all user profiles' needs. At the age of 20, the JASPAR team looks ahead to strengthen its contribution within the open science ecosystem to which it contributed, consolidating and supporting the field in deepening our understanding of the role of TF binding in gene regulation.

## Supplementary Material

gkad1059_Supplemental_File

## Data Availability

JASPAR is freely available at https://jaspar.elixir.no/.
