# Supplementary material for: JASPAR 2024: 20th anniversary of the open-access database of transcription factor binding profiles
Source: Nucleic Acids Res. 2023 Nov 14;52(D1):D174–82. doi: 10.1093/nar/gkad1059 (PMC10767809; doi:10.1093/nar/gkad1059)
Supplement: gkad1059_Supplemental_File [file gkad1059_supplemental_file.pdf]

JASPAR 2024: 20th anniversary of the open-access database of transcription factor binding profiles

Ieva Rauluseviciute[1,*], Rafael Riudavets Puig[1,*], Romain Blanc-Mathieu[2,+], Jaime A. Castro-Mondragon[1,+], Katalin Ferenc[1,+], Vipin Kumar[1,+], Roza Berhanu Lemma[1,+], Jérémy Lucas[2,+], Jeanne Chèneby[3], Damir Baranasic[4,5,6], Aziz Khan[1,7], Oriol Fornes[8], Sveinung Gundersen[3], Morten Johansen[3], Eivind Hovig[3,9], Boris Lenhard[4,5,§], Albin Sandelin[10,§], Wyeth W. Wasserman[8,§], François Parcy[2,§], Anthony Mathelier[1,11,§]

[1] Centre for Molecular Medicine Norway (NCMM), Nordic EMBL Partnership, University of Oslo, 0318 Oslo, Norway
[2] Laboratoire Physiologie Cellulaire et Végétale, Univ. Grenoble Alpes, CNRS, CEA, INRAE, IRIG-DBSCI-LPCV, 17 avenue des martyrs, F-38054, Grenoble, France
[3] Center for Bioinformatics, Department of Informatics, University of Oslo, Oslo, Norway
[4] MRC London Institute of Medical Sciences, Du Cane Road, London, W12 0NN, UK
[5] Institute of Clinical Sciences, Faculty of Medicine, Imperial College London, Hammersmith Hospital Campus, Du Cane Road, London, W12 0NN, UK
[6] Division of Electronics, Ruđer Bošković Institute, Bijenička cesta, 10000 Zagreb, Croatia
[7] Stanford Cancer Institute, Stanford University School of Medicine, Stanford, California, 94305, USA
[8] Centre for Molecular Medicine and Therapeutics, Department of Medical Genetics, BC Children's Hospital Research Institute, University of British Columbia, 950 W 28th Ave, Vancouver, BC V5Z 4H4, Canada
[9] Department of Tumor Biology, Institute for Cancer Research, Oslo University Hospital, 0424 Oslo, Norway
[10] Department of Biology and Biotech Research and Innovation Centre, University of Copenhagen, Ole Maaløes Vej 5, DK2200 Copenhagen N, Denmark
[11] Department of Medical Genetics, Institute of Clinical Medicine, University of Oslo and Oslo University Hospital, Oslo, Norway

[*] These authors contributed equally to this work as co-first authors
[+] These authors contributed equally to this work as co-second authors
[§] To whom correspondence should be addressed: anthony.mathelier@ncmm.uio.no; francois.parcy@cea.fr; wyeth@cmmt.ubc.ca; albin@binf.ku.dk; b.lenhard@imperial.ac.uk

SUPPLEMENTARY TEXT

Data processing for manual curation

The data processed for this release is provided in Supplementary Table S1 (1–9). PFMs enriched in the datasets were obtained as described previously (Supplementary Text of the JASPAR 2022 manuscript (4)). The pipeline code used to process data for this release is available at https://bitbucket.org/CBGR/jaspar_curation_pipeline/src/JASPAR2024/.

Motif trimming

To trim JAPAR PFMs, we first identified consecutive positions with an IC < 0.3, as previously used in the literature (10–12), starting from the edges of the PFMs. Next, we identified IC "spikes", which are positions with IC > 0.3 directly surrounded by upstream and downstream positions with IC < 0.3. If this "spike" position has an IC < 0.6, it is trimmed with the surrounding position with IC < 0.3, as identified from the edges. Motif trimming is part of the data preparation for manual curation pipeline as a separate workflow and the particular script that was used to perform trimming is available at https://bitbucket.org/CBGR/jaspar_curation_pipeline/src/JASPAR2024/bin/matrix-clustering_stand-alone/convert-matrix.R. The trimming workflow, which includes summaries of trimmed positions and a

comparison of Gini coefficients for trimmed and untrimmed profiles, is available at https://bitbucket.org/CBGR/jaspar_curation_pipeline/src/main/trim_profiles/trim_profiles_workflow.Rmd.

Matrix clustering

We clustered and aligned PFMs from the CORE and the CORE+UNVALIDATED collections in six taxa (fungi, insects, nematodes, plants, urochordates and vertebrates). Specifically, we developed an updated stand-alone version of the RSAT *matrix-clustering* tool (13) (https://github.com/jaimicore/matrix-clustering_stand-alone). The clustered PFMs are computed and visualised as radial (all motifs aligned) and linear trees (cluster alignment) using the following parameters: `"-m “Ncor” -W 4 -n 0.5"`. We used the structural annotation of the TFs to annotate the radial trees. This tool was integrated into our workflow as a container and is available at https://hub.docker.com/repository/docker/cbgr/matrix_clustering/general.

Complementary data analysis

Sequence logos, word clouds, familial binding profiles, genomic tracks, TFFMs, and LOLA databases were computed as described before (Supplementary Text of JASPAR 2022 manuscript (4)). All analyses are now integrated into a single workflow available at https://bitbucket.org/CBGR/jaspar_2024_downstream/src/main/. Word clouds, TFFMs, TFBS, and LOLA computation are available as containers at https://hub.docker.com/repositories/cbgr. The LOLA databases are available as RDS R objects on Zenodo at https://doi.org/10.5281/zenodo.8341374.

Software development

To prepare the new release of the JASPAR database, we rely on “3D” software components - **d**iscovery, **d**ownstream, **d**eployment. In more detail:

1) The *discovery* pipeline analyses peak data by discovering TF motifs or processes external PFM data and prepares everything for manual curation. The code is available at https://bitbucket.org/CBGR/jaspar_curation_pipeline/src/JASPAR2024/.
2) The *downstream* analysis pipeline uses manually curated PFM data, processes, and prepares the database update. The code is available at https://bitbucket.org/CBGR/jaspar_2024_downstream/src/jaspar2024/.
3) The *deployment* pipeline deploys the updated database into the website. The code is available at https://bitbucket.org/CBGR/jaspar2020/src/jaspar2024/.

All three processes received major updates with this release to increase software quality and reproducibility. Data processing for the manual curation pipeline was expanded to add motif trimming (described above). The downstream analysis pipeline was designed to process manually curated profiles of the 2024 release to ensure future maintenance and the integration of new features. We have put a strong emphasis on good software development practices, including continuous integration, containerisation of external resources (https://hub.docker.com/repositories/cbgr), version control, code reviews, extensive documentation, automated checks, and unit tests to ensure good quality data processing code. This effort aligns with the core principle of high-quality resources provided in JASPAR, as we believe better software is the way to achieve better research (14). The website

deployment used Ansible (https://docs.ansible.com/ansible/latest/getting_started/index.html) and Jenkins (https://www.jenkins.io/) as the workflow management solution. ELIXIR Norway now hosts the JASPAR website on Norwegian Research and Education Cloud (NREC) resources. The Ansible playbook code is available a https://github.com/elixir-oslo/jaspar-playbook.

Retrieving JASPAR paper citations

The citations for the nine JASPAR papers were retrieved using the R package `scholar` (https://github.com/jkeirstead/scholar) to query Google Scholar.

References

1. Kolmykov,S., Yevshin,I., Kulyashov,M., Sharipov,R., Kondrakhin,Y., Makeev,V.J., Kulakovskiy,I.V., Kel,A. and Kolpakov,F. (2021) GTRD: an integrated view of transcription regulation. *Nucleic Acids Res.*, **49**, D104–D111.

2. Weirauch,M.T., Yang,A., Albu,M., Cote,A.G., Montenegro-Montero,A., Drewe,P., Najafabadi,H.S., Lambert,S.A., Mann,I., Cook,K., *et al.* (2014) Determination and inference of eukaryotic transcription factor sequence specificity. *Cell*, **158**, 1431–1443.

3. Lai,W.K.M., Mariani,L., Rothschild,G., Smith,E.R., Venters,B.J., Blanda,T.R., Kuntala,P.K., Bocklund,K., Mairose,J., Dweikat,S.N., *et al.* (2021) A ChIP-exo screen of 887 Protein Capture Reagents Program transcription factor antibodies in human cells. *Genome Res.*, **31**, 1663–1679.

4. Castro-Mondragon,J.A., Riudavets-Puig,R., Rauluseviciute,I., Lemma,R.B., Turchi,L., Blanc-Mathieu,R., Lucas,J., Boddie,P., Khan,A., Manosalva Pérez,N., *et al.* (2022) JASPAR 2022: the 9th release of the open-access database of transcription factor binding profiles. *Nucleic Acids Res.*, **50**, D165–D173.

5. Ricci,W.A., Lu,Z., Ji,L., Marand,A.P., Ethridge,C.L., Murphy,N.G., Noshay,J.M., Galli,M., Mejía-Guerra,M.K., Colomé-Tatché,M., *et al.* (2019) Widespread long-range cis-regulatory elements in the maize genome. *Nat Plants*, **5**, 1237–1249.

6. Pei,H., Teng,W., Gao,L., Gao,H., Ren,X., Liu,Y., Jia,J., Tong,Y., Wang,Y. and Lu,Z. (2023) Low-affinity SPL binding sites contribute to subgenome expression divergence in allohexaploid wheat. *Sci. China Life Sci.*, **66**, 819–834.

7. Xu,Z., Casaretto,J.A., Bi,Y.-M. and Rothstein,S.J. (2017) Genome-wide binding analysis of AtGNC and AtCGA1 demonstrates their cross-regulation and common and specific functions. *Plant Direct*, **1**, e00016.

8. Liu,S., Kracher,B., Ziegler,J., Birkenbihl,R.P. and Somssich,I.E. (2015) Negative regulation of ABA signaling by WRKY33 is critical for Arabidopsis immunity towards Botrytis cinerea 2100. *Elife*, **4**, e07295.

9. Fuxman Bass,J.I., Pons,C., Kozlowski,L., Reece-Hoyes,J.S., Shrestha,S., Holdorf,A.D., Mori,A., Myers,C.L. and Walhout,A.J. (2016) A gene-centered C. elegans protein-DNA interaction network provides a framework for functional predictions. *Mol. Syst. Biol.*, **12**, 884.

10. Mahony,S. and Benos,P.V. (2007) STAMP: a web tool for exploring DNA-binding motif similarities. *Nucleic Acids Res.*, **35**, W253–8.

11. Broin,P.Ó., Smith,T.J. and Golden,A.A. (2015) Alignment-free clustering of transcription factor binding motifs using a genetic-k-medoids approach. *BMC Bioinformatics*, **16**, 22.

12. Gordân,R., Murphy,K.F., McCord,R.P., Zhu,C., Vedenko,A. and Bulyk,M.L. (2011) Curated collection of yeast transcription factor DNA binding specificity data reveals novel structural and gene regulatory insights. *Genome Biol.*, **12**, R125.

13. Castro-Mondragon,J.A., Jaeger,S., Thieffry,D., Thomas-Chollier,M. and van Helden,J. (2017) RSAT matrix-clustering: dynamic exploration and redundancy reduction of transcription factor binding motif collections. *Nucleic Acids Res.*, **45**, e119.

14. Goble,C. (2014) Better Software, Better Research. *IEEE Internet Comput.*, **18**, 4–8.

SUPPLEMENTARY FIGURES

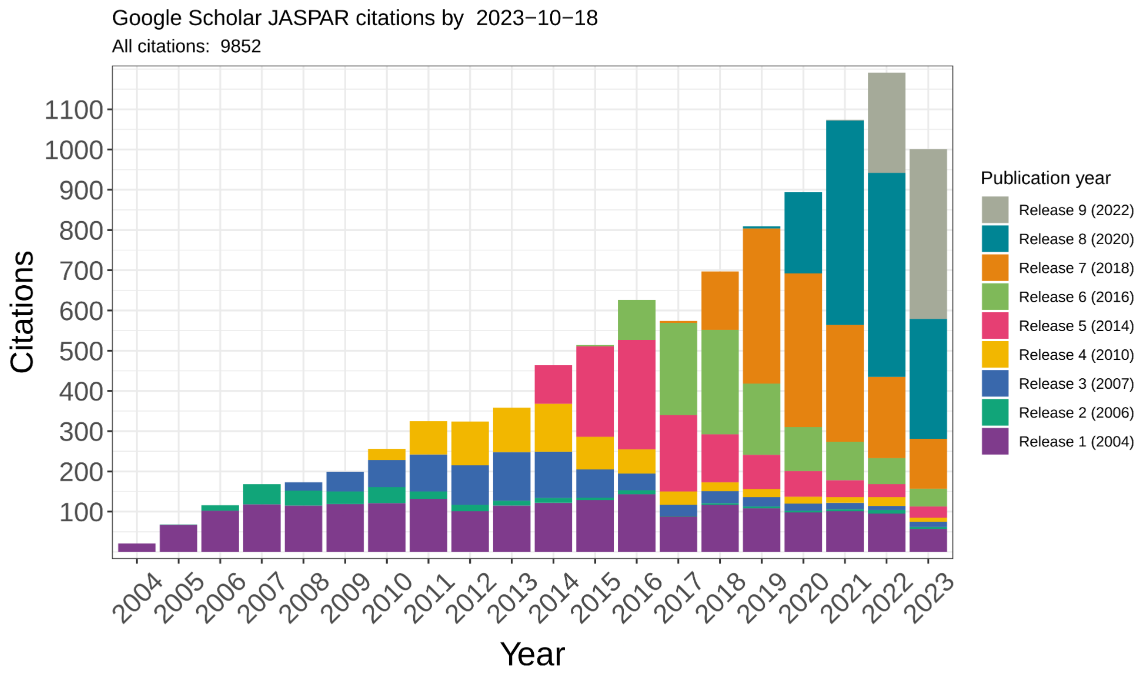

Supplementary Figure S1. JASPAR database papers citations over the years. The number of citations for each JASPAR release paper over the years since JASPAR's first release in 2004. Citation information from Google Scholar (last updated on 2023-10-18).

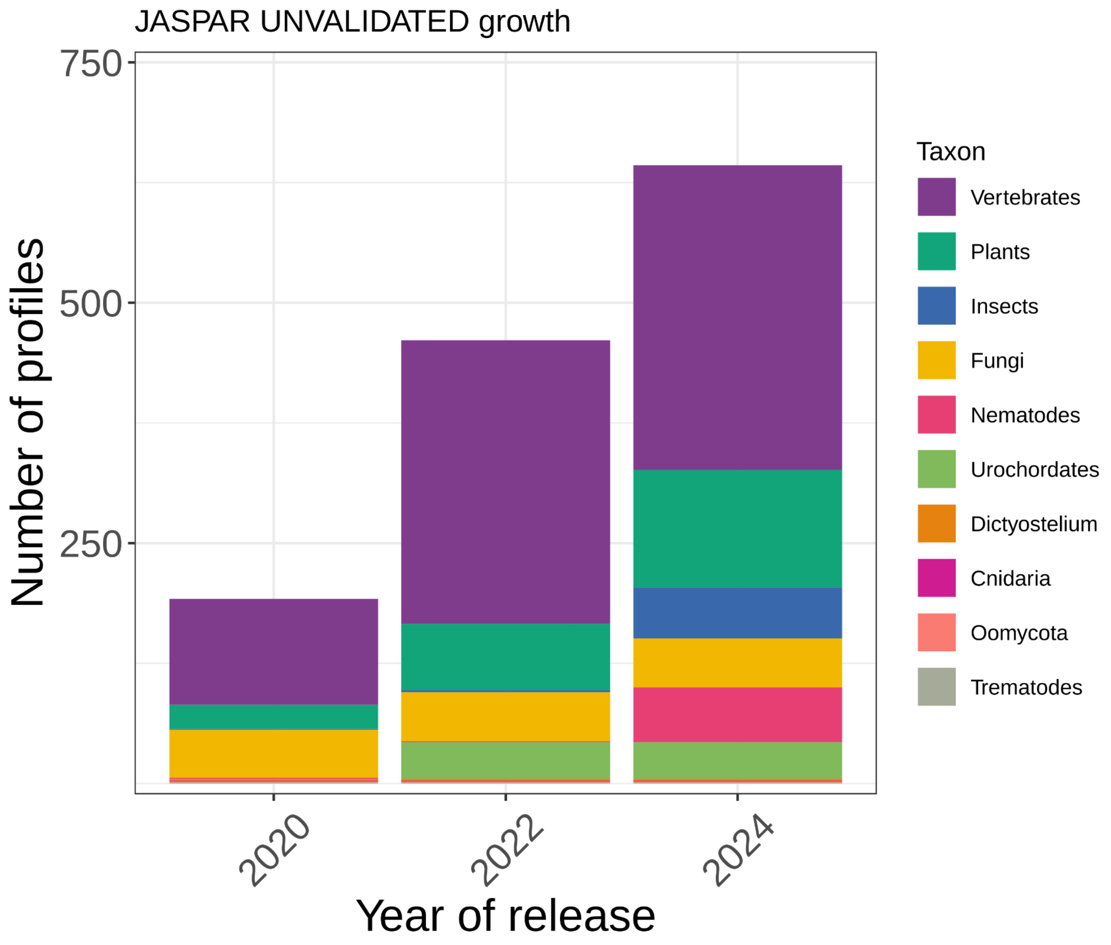

Supplementary Figure S2. JASPAR UNVALIDATED collection growth. The number of profiles for each taxon in UNVALIDATED collection every release since its introduction in the JASPAR 2020 (8th release).

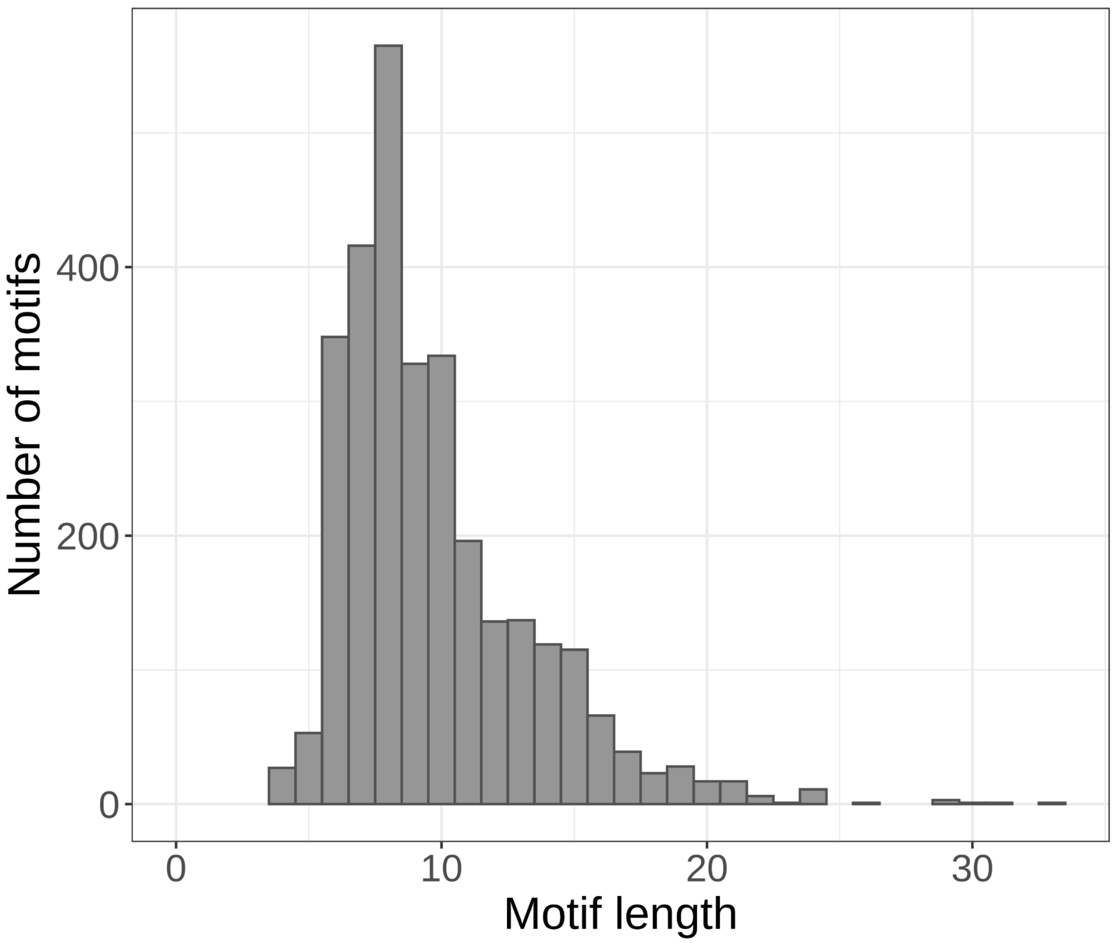

Supplementary Figure S3. Motif size distribution for CORE and UNVALIDATED profiles in JASPAR 2024. Matrix sizes of JASPAR matrices vary from 4 to 33 base pairs.

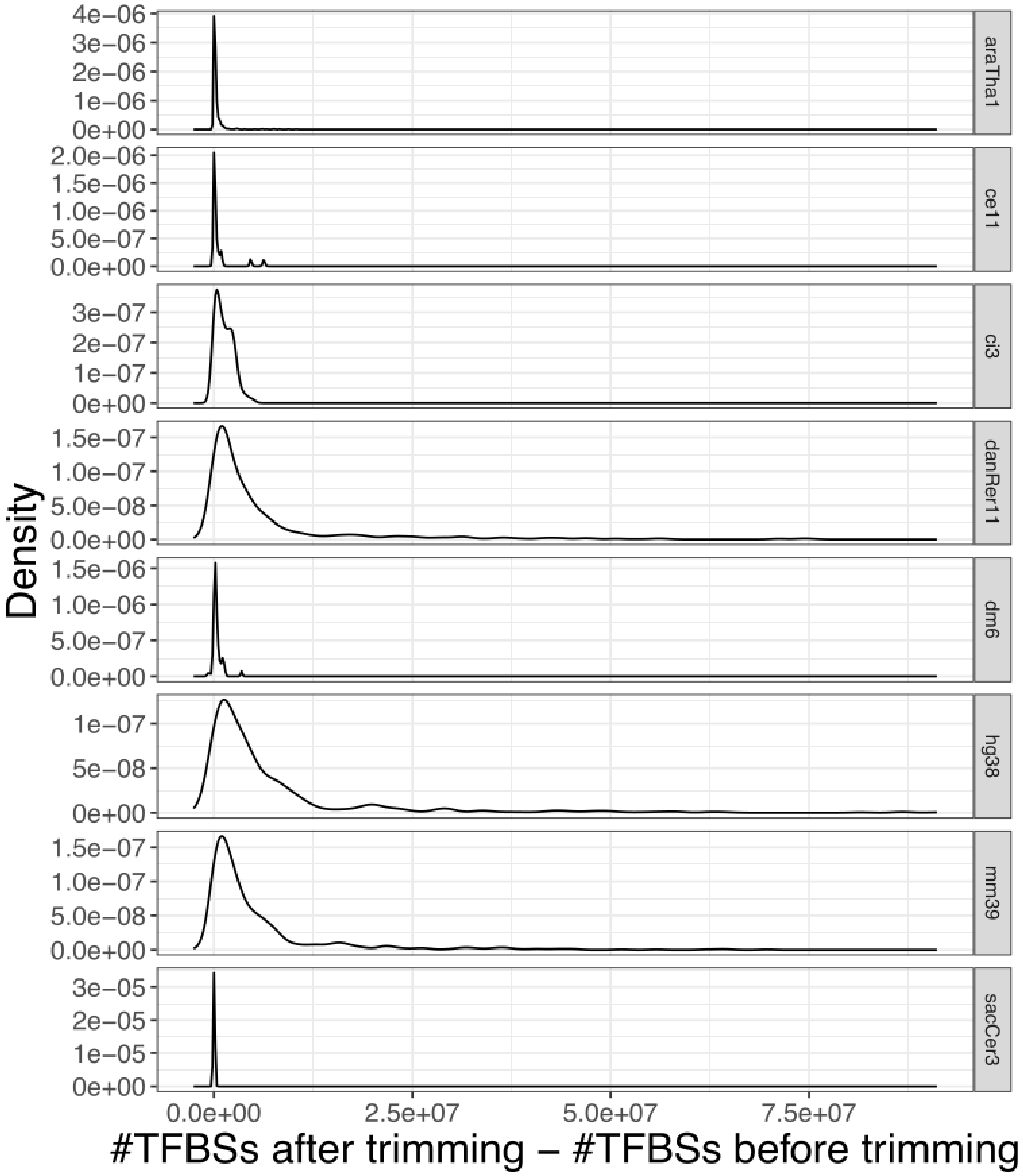

Supplementary Figure S4. Distribution of the change in the number of TFBS predictions before and after trimming. For each trimmed profile, the x-axis shows the difference in the number of TFBS predictions after trimming (from JASPAR 2024) minus the number of predictions before trimming (from JASPAR 2022).

SUPPLEMENTARY TABLES

Supplementary Table S1. Data processed for the 10th JASPAR release (JASPAR 2024). The table contains information on data type, the corresponding organism on which the data is generated and the source of the data.

**Source**	**Organism**	**Data type**	**Number of motifs generated/ retrieved**	**Reference**
GTRD	*D. rerio*	ChIP-seq	42	PMID: 33231677
	C. elegans		1,398	
	D. melanogaster		2,937	
	A. thaliana		369	
	H. sapiens		10,206	
	R. norvegicus		153	
	M. musculus		7,035	
	S. cerevisiae		1,562	
	S. pombe		305	
CIS-BP	*D. melanogaster*	B1H	593	PMID: 25215497
		ChIP-seq		
		PBM		
		SELEX		
	C. elegans	ChIP-seq	173	
		PBM		
	Plants	PBM	521	
		DAP-seq		
Lai *et al.*	*H. sapiens*	ChIP-exo	778	PMID: 34426512
		PBM	12	
JASPAR 2022 UNVALIDATED collection	Vertebrates	Collection of data types	1,167	PMID: 34850907
	Nematodes		45	
	Insect		156	

	Fungi		231	
	Urochordates		132	
	Plants		769	
Ricci *et al.*	*Z. mays*	DAP-seq	51	PMID: 31740773
Cerise *et al.*	*O. sativa*	DAP-seq	9	GSE207402
Pei *et al.*	*T. aestivum*	DAP-seq	117	PMID: 36417050
Xu *et al.*	*A. thaliana*	ChIP-seq	12	PMID: 31245665
Liu *et al.*	*A. thaliana*	ChIP-seq	6	PMID: 26076231
Bass *et al.*	*C. elegans*	Y1H	350	PMID: 27777270

Supplementary Table S2. Numbers of profiles in JASPAR taxon-specific collections over different releases.

Taxon	Collection	2004	2006	2008	2010	2012	2014	2016	2018	2020	2022	2024
Vertebrates	CORE	79	87	96	125	125	200	510	570	739	840	879
	UNVALIDATED									110	295	317
Plants	CORE	13	15	17	17	17	58	215	459	501	649	805
	UNVALIDATED									25	69	122
Insects	CORE	13	13	14	122	122	130	133	133	143	149	286
	UNVALIDATED									1	2	53
Fungi	CORE				170	170	170	171	171	178	178	178
	UNVALIDATED									50	51	51
Nematodes	CORE				5	5	14	25	25	42	42	103
	UNVALIDATED									1	1	57
Urochordates	CORE		1	1	1	1	1	1	1	1	86	94
	UNVALIDATED										38	38
Diatoms	CORE								1	1	1	1
Trematodes	UNVALIDATED									1	1	1
Oomycota	UNVALIDATED									1	1	1
Dictyostelium	UNVALIDATED									2	2	2
Cnidaria	UNVALIDATED									1	1	1

Supplementary Table S3. Overview of the JASPAR 2024 UNVALIDATED collection update compared to the JASPAR 2022 UNVALIDATED collection.

**Taxonomic group in UNVALIDATED collection**	**Non-redundant PFMs in JASPAR 2022**	**New non-redundant PFMs in JASPAR 2024**	**Removed profiles**	**Upgraded profiles (from UNVALI-DATED to CORE)**	**Updated PFMs in JASPAR 2024**	**Total non-redundant PFMs in JASPAR 2024**
Plants	113	53	2	42	-	122
Vertebrates	326	22	11	20	-	317
Urochordata	46	-	-	8	-	38
Insects	6	51	2	2	-	53
Nematodes	2	56	1	-	-	57
Fungi	52	-	1	-	-	51
Dictyostelium	2	-	-	-	-	2
Cnidaria	1	-	-	-	-	1
Trematodes	1	-	-	-	-	1
Oomycota	1	-	-	-	-	1
**UNVALIDATED total**	**550**	**182**	**17**	**72**	**-**	**643**

Supplementary Table S4. Overview of the JASPAR 2024 TFFM collection.

**Taxonomic group**	**Number of TFFMs**
Plants	423
Vertebrates	648
Insects	35
Nematodes	24
Fungi	5
**Total**	**1,135**
